# Evaluation of a clinical decision support design guideline

**DOI:** 10.1093/jamia/ocag138

**Published:** 2026-08-27

**Authors:** Selvana Awad, Thomas Loveday, Andrew Baillie, Melissa T Baysari

**Affiliations:** Faculty of Medicine and Health, The University of Sydney, Australia; eHealth NSW, Australia; eHealth NSW, Australia; Faculty of Medicine and Health, The University of Sydney, Australia; Faculty of Medicine and Health, The University of Sydney, Australia

**Keywords:** human factors, patient safety, health information technology, clinical decision support

## Abstract

**Objectives:**

Human Factors (HF) principles are essential for safe and effective clinical decision support (CDS), yet existing guidance is fragmented and rarely evaluated in real world settings. A novel, evidence-based, vendor-agnostic HF-informed guideline was developed to address this gap. This study evaluated its perceived usefulness, usability, and impact on CDS design and optimization.

**Materials and Methods:**

A 2-phase explanatory sequential mixed methods approach was used. Phase 1 involved a survey of guideline users (*n* = 25) assessing usefulness, ease of use, satisfaction, and influence on decision-making. Phase 2 included semi-structured interviews (*n* = 10) to explore real-world application experiences. Quantitative data were analyzed descriptively, and qualitative data were analyzed thematically using a general inductive approach.

**Results:**

Findings were consistently positive. Participants reported that the guideline was easy to use and supported structured, evidence-based CDS decision-making. It improved confidence in selecting and designing CDS interventions, with particular value placed on its consolidated format and step-by-step approach. Suggested enhancements included adding practical tools, worked examples, and case studies to support broader application across contexts.

**Discussion:**

This study represents the first real-world evaluation of a consolidated HF-informed CDS guideline, addressing the gap between theory and practice. Results indicate that integrated, accessible HF guidance can strengthen consistency and quality in CDS design, particularly when embedded within routine workflows and governance processes.

**Conclusion:**

The guideline is a valuable, practical resource for CDS design and optimization. Future research should assess its impact on post-implementation optimization and include objective measures of usability, safety, and CDS effectiveness to further demonstrate its value.

## Background and significance

Clinical decision support (CDS) in health information technology (HIT) provides health care workers and patients with relevant knowledge, context, and person-specific information, at appropriate times to support care delivery.^1^ Well-designed CDS can support the delivery of safe, quality patient care, however, inappropriate or poorly designed CDS can introduce usability issues and potentially contribute to harm by creating alert fatigue, increasing cognitive overload, and slowing down the HIT system.^2^^,^^3^

Human factors (HF) or ergonomics explores the interaction between humans and systems within the work system.^4^ HF is used across many safety critical industries to support the design of work systems which consist of the people, tasks, tools/technologies, the physical environment, and organizational factors that influence how work is performed.^4^ Although the application of HF to healthcare has increased steadily over the past decade, the use of HF to support the design of CDS is variable and can be optimized.^5^

Alert fatigue is a well reported phenomenon in HIT. It occurs when excessive or inappropriate interruptive alerts are implemented resulting in alerts, including potentially important alerts, being ignored by users.^6^ While established HF informed standards for alerts and alarms are routinely used in other industries such as aviation, this is not common practice in HIT.^5^^,^^7–9^ Earlier CDS guidance, including the *Ten Commandments for Effective Clinical Decision Support*, established several enduring principles for CDS design, such as integration into clinical workflow and provision of support at the time and location of decision-making.^10^ However, guidance on optimal CDS design including alerts in the context of HIT is spread across multiple sources, for example various design checklists,^11^^,^^12^ or is outdated due to predating major advances in decision support technologies. For example, the Osheroff et al.^13^ guideline has not been updated since 2012 and is not accessible without payment.

In an earlier paper, we describe the development and initial validation of a statewide, best practice HF informed CDS design guideline, developed in response to overreliance on interruptive alerts resulting in alert fatigue, and the lack of structure and process around CDS decision making in our context.^8^ Ineffective CDS governance processes can result in unstructured and opinion-based CDS discussions, leading to suboptimal decision making.^14^ With plans to configure and implement the Single Digital Patient Record (SDPR) in NSW, a major health transformation program involving the implementation of a new patient administration system, electronic medical record, and laboratory information system across all NSW public hospitals; the need for effective statewide CDS moving forward was recognized.^8^ This provided the impetus to develop an HF-informed, accessible and consolidated best practice CDS design guideline that could provide evidence-based structure to CDS discussions for both current state legacy systems as well as new systems being designed.^8^ The guideline is the first of its kind to consolidate best practice knowledge from various sources including industry standards and the latest literature to provide practical guidance on the optimal selection and design of CDS within the HIT context.^8^

The guideline underwent preliminary evaluation during its development including testing sections of the guideline with HF experts,^8^ and it was released in September 2024. This paper extends that work to evaluate the guideline’s practical utility, usability, and application to inform decision making about CDS in practice.^7^

## Objectives

This research project aimed to understand how a CDS Design Guideline is being used in the context of real-world projects and use cases following its release; evaluate its usefulness, usability and impact; and identify strategies to support its implementation.

## Materials and methods

### Design

This was an explanatory sequential mixed methods study adopting 2 phases: (1) an online survey and (2) in-depth interviews. The Consensus-Based Checklist for Reporting of Survey Studies (CROSS) and Consolidated Criteria for Reporting Qualitative Research (COREQ) Checklist were used to guide manuscript preparation.^15^^,^^16^

### The CDS guideline

The vendor-agnostic, HF-based guideline was developed to guide the design, evaluation, and continuous improvement of CDS throughout its lifecycle.^8^ The guideline consolidates recommendations from HF standards, literature, and best-practice guidance into a practical resource for clinicians, informatics professionals, system designers, and governance groups involved in CDS decision-making.^8^ It consists of 4 sections: (1) an overview of the guideline and HF principles relevant to CDS; (2) guidance to determine whether CDS is appropriate, including consideration of the underlying problem, alternative interventions, and selection of suitable CDS options; (3) CDS governance and review processes, including lifecycle management considerations; and (4) detailed design guidance for specific CDS interventions. The guideline includes practical tools such as decision-making flowcharts, a CDS decision matrix, alert rationalization templates, guidance on passive and interruptive CDS, and prompts to support consideration of workflow, usability, and patient safety factors. The guideline differs from previous CDS resources by providing a consolidated, HF-informed approach that supports not only the design of CDS interventions, but also decisions regarding whether CDS is required, governance processes, evaluation, optimization, and lifecycle management.^8^ Key sections of the guideline can be found in a previous paper that describes its development.^8^ The guideline is currently available from the corresponding author upon reasonable request.

Existing CDS frameworks, such as the CDS “Five Rights,” provide high-level principles for delivering the right information to the right person, in the right intervention format, through the right channel, at the right point in workflow, and at the right time.^13^^,^^17^ However, these frameworks provide limited guidance on determining whether CDS is the most appropriate solution to a problem, selecting among CDS options, governing CDS decisions, or evaluating and optimizing CDS over time. The guideline evaluated in this study was developed to address these gaps by consolidating HF standards, literature, and best-practice recommendations into a practical resource that supports the full CDS lifecycle. The guideline is informed by HF concepts including human-centered design, systems thinking, workflow integration, usability, cognitive support, alert fatigue mitigation, and continuous improvement. Its novelty lies in the integration of these HF principles with governance, decision making, design, evaluation, and optimization guidance within a single, vendor-agnostic resource that can be applied by HF and non-HF experts.

### Phase 1

The survey instrument was developed by 2 researchers with expertise in HF (S.A. and M.T.B.), and informed by usability concepts such as effectiveness, efficiency, and satisfaction.^18^ The survey comprised 5 sections and 16 items, including questions which collected demographic information, data on the guideline’s usefulness, ease of use, understandability, satisfaction and impact, using 5-point Likert scales, and free-text comments (Appendix A). The survey was reviewed by a senior researcher in implementation science (A.B.) and a senior human factors specialist (T.L.), prior to being pilot tested by a junior researcher external to the research team (J.L.) to ensure questions were understandable and flowed well. The survey was administered online using the Qualtrics platform^6^ and was open for a 3-month period (October to December 2024).

### Phase 2

To complement the survey, and allow a more in-depth understanding of the guideline’s use, impact, and implementation, semi-structured interviews were held via videoconferencing with those involved in CDS decision making and governance processes. Questions used to guide the semi-structured interviews (Appendix B) were developed by S.A. and reviewed by another member of the research team (M.T.B.). Interviews were conducted by S.A. and M.T.B. All interviews were audio-recorded and transcribed.

### Recruitment

The recruitment strategy aimed to invite anyone who was in a role where the guideline could have been used to participate in the survey. This was done through a purposive sampling method in conjunction with snowball (word of mouth) sampling. Participants were recruited through direct emails, and advertising via organizational forums and channels (eg, presentations at governance groups and newsletters) within NSW organizations and national societies.

Reminders to complete the survey were sent out twice; at the start of the survey period and 1 month before the survey’s closing date. Participants who were interested in participating in a follow-up interview were asked to express their interest and leave their contact details at the end of the survey. In addition, participants who were known users of the guideline or expected to be users based on the research team’s knowledge, were directly approached and invited to take part in an interview. For the interviews, recruitment ceased when no new themes or concepts were identified in successive interviews, indicating thematic saturation.

Surveys and interviews were voluntary and no compensation was provided.

### Data analysis and reporting

Survey data were analyzed using descriptive statistics. Free text responses were reviewed by 2 researchers, inductively coded, and then codes were synthesized into themes via a discussion process.

Similarly, interview data were de-identified and independently thematically analyzed by 2 researchers (S.A. and M.T.B.) using a general inductive approach.^19^ Each investigator initially familiarized themselves with 3 transcripts and undertook open coding, assigning codes to segments of text that captured key concepts related to guideline use, usability, usefulness, impact, and implementation. Codes were developed inductively from the data rather than being derived from a preexisting framework. Following independent coding, the researchers met to compare and discuss codes. Related codes were then grouped into higher-order categories and synthesized into broader themes through an iterative process of discussion and refinement. Any discrepancies in coding or theme development were resolved through discussion until consensus was reached. One of the investigators then coded the rest of the interviews using the agreed themes and codes. Representative quotations were selected to illustrate each theme.

### Ethics and consent

Ethics approval was obtained from the University of Sydney’s Human Research Ethics Committee. All participants who took part in the online survey provided written informed consent by answering a question in the survey tool. Interview participants provided written consent via an online consent form administered via Qualtrics.

## Results

### Information on survey respondents and interview participants

#### Survey respondents

Twenty-five participants responded to the survey and 24 provided informed consent for their responses to be used. Six participants indicated that they were interested in participating in a follow-up interview and left their contact details at the end of the survey.

Appendix C summarizes respondents’ reported primary area of work while using the CDS Design Guideline. “Informatics/digital health” was the most frequently reported work area where the guideline was used (45.8% of 24 respondents), followed by “clinical governance/patient safety” (20.8% of 24 respondents).

#### Interview participants

Ten participants were interviewed and interviews lasted on average 35.4 minutes (range 22.5–52.5 minutes). Three of the interview participants had also completed the survey. Of the 10 participants, 9 were trained as pharmacists and 1 as a medical doctor: 6 worked in informatics, 2 worked in informatics as well as management, and 1 worked in safety and quality. All participants were from Australia, except 1 who was from the United States.

### Use of the guideline

Seventeen (71%) of the 24 survey participants reported using the guideline; however, not all questions were answered by these participants. The main reason reported for not using the guideline was lack of awareness: *“I was unaware of this guideline.”* [PS8]

Table 1 summarizes the purpose/s for which the CDS Design Guideline was reportedly used by the 15 survey respondents who answered the question. Based on the survey, the guideline was mainly used to inform decision making about CDS (eg, whether an alert should be introduced), support CDS discussions (eg, to guide configuration discussions), and served as an educational tool or reference material (eg, to access required information). Most interview participants also reported that governance groups used the guideline to guide decision making and inform their processes around CDS, such as their intake process for receiving requests for alerts: *“the other thing we really liked about this guideline that we’re currently working into our intake process is actually that table that about the, you know, the five rights of clinical decision support, like where do you want it, who do you want it and so on and so forth…that has been extremely helpful because it does help you really understand.”* [P4]

**Table 1.** Purpose/s for which the CDS design guideline was reported to be used based on the survey and interviews.

Purpose	No. of responses from the survey	Quote/s from the survey (PS) and interviews (P), where available
Inform decision making about CDS and relevant processes	12	*“to assess if an alert should be built or not”* [PS15] *“The guideline has been very effective to guide best practice SDPR configuration. It has assisted with efficient decision making and improved awareness of human factors”* [PS19] *“Currently using to evaluate if we downgrade drug interaction checking for pharmacists.”* [PS6] *“there’s a clear guide to follow when we’re making decisions, and often you know when you’ve got 12 people with strong opinions and type A personalities in a room… having a guide to follow helps me as a chair to …help the decision-making be functional and clear and bring it back to us”* [P9]
Provide advice about CDS	6	*N/A*
Support CDS discussions	12	*“to justify changes required for pre-existing alert designs”* [PS15] *“It supported discussions around a new CDS request and helped to logically explore the problem and alternative options to address the issue other than CDS.”* [PS17] *“we follow the guideline…we sort of follow that through our decisions… and when we’re having a debate as a group or struggling to make a decision, we bring it back to the guideline and our [organisational] risk matrix and try and figure it out…”* [P10]
As an education tool or reference material	11	*“to compare pre-existing alert designs against”* [PS15] *“I searched for the specific information I needed after a brief review of the document.”* [PS15]

Abbreviations: CDS, clinical decision support; SDPR, Single Digital Patient Record.

Figures 1 and 2 describe more detailed, specific examples of how the guideline was used to inform decision making about CDS based on the in-depth interviews.

**Figure 1.**
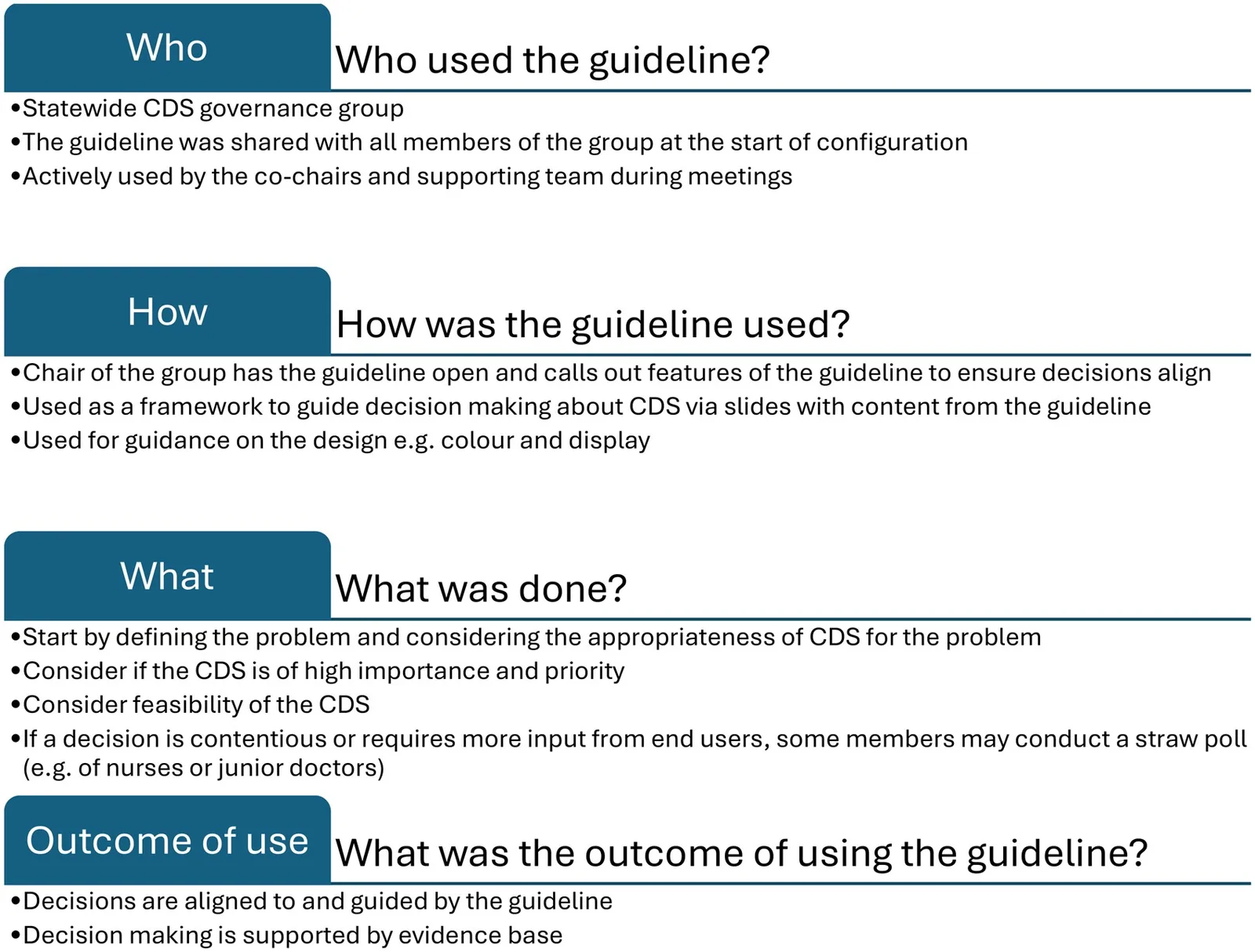
Example of how the guideline was used by a statewide clinical decision support (CDS) governance group.

**Figure 2.**
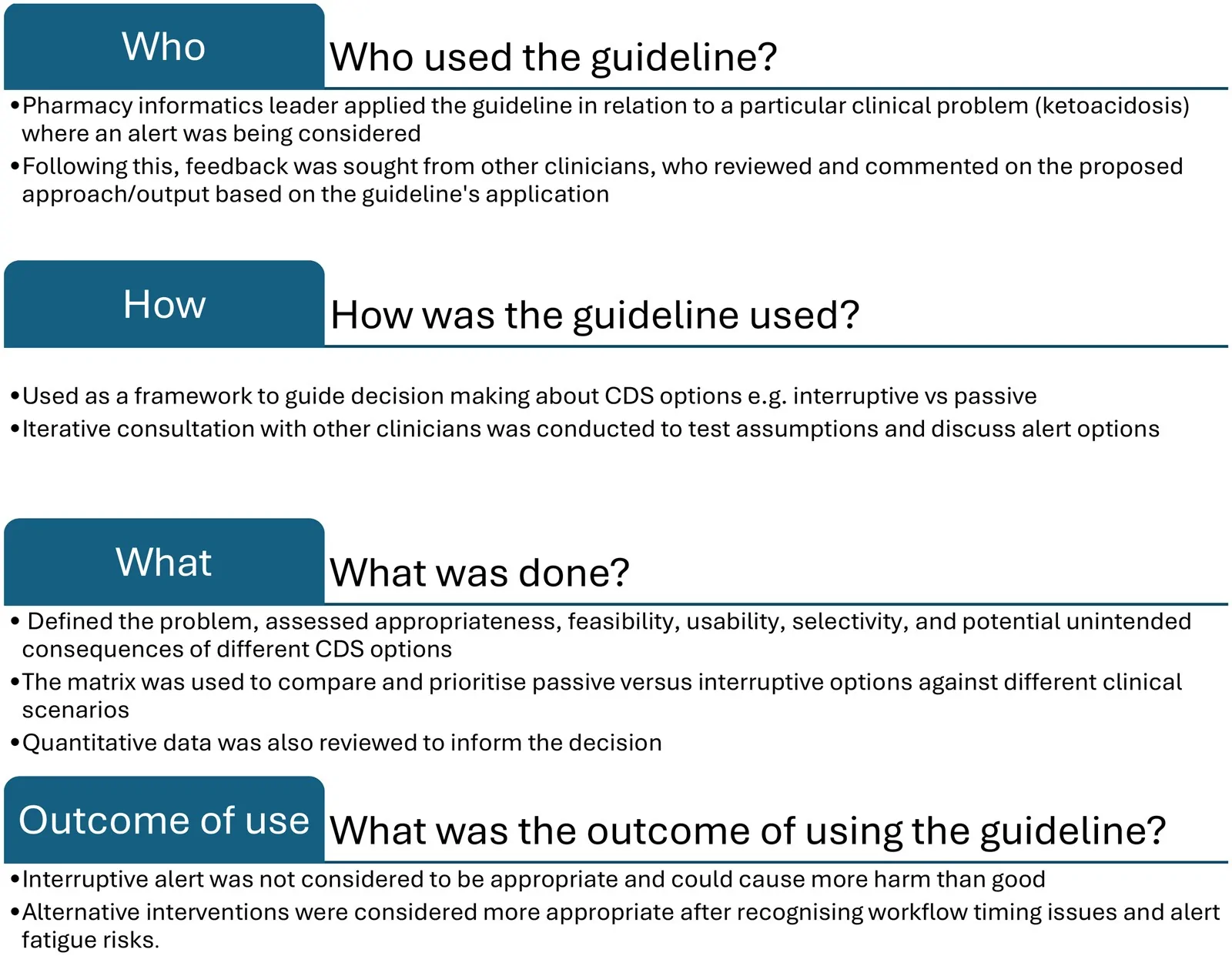
Example of how the guideline was used by an informatics professional.

Based on the survey, the number of times the guideline had been used varied (see Figure 3). Responses from 15 respondents of the survey indicated that all sections of the guideline were used, with sections 2 and 4 most frequently used (12 and 11 counts, respectively), followed by section 3 and section 1 (6 and 5 counts, respectively).

**Figure 3.**
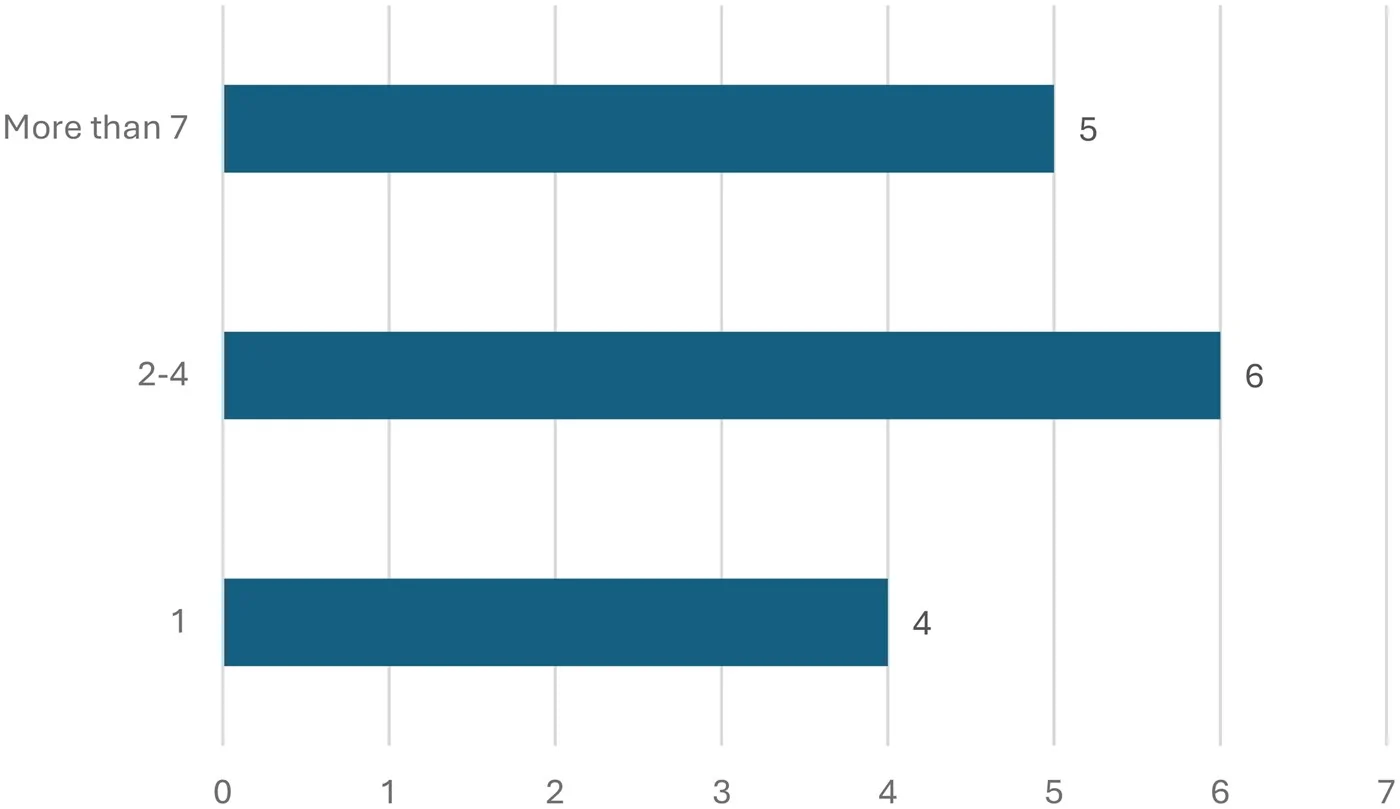
Number of use cases/situations the clinical decision support (CDS) Design Guideline was reportedly used for.

Survey respondents indicated that the guideline was used to support the design of new systems and the redesign of old systems: *“extensively in SDPR design working group [for future systems being configured] but [is] also useful for current and legacy decision making and prioritisation”* [PS16].

Interview participants provided more detail on how the guideline was used and reported using the guideline in a way that was contextualized to their local settings, systems, and processes. For example, some participants used the guideline to develop their own local procedure on alerts specifically targeted at clinicians: *“we really need to create a local framework or policy or clinical procedure where we help everyone else understand the background behind alerts and how we wish to use them as an organisation. And that’s where we leverage the NSW framework to create a custom one that’s more specific to our organisation.”* [P3]

How the guideline was used also varied depending on the purpose of use or task: *“I found the relevant section and I read through the details and I made sure that when we were workshopping what the text would say, that we were referring back to the guidance from the document.”* [P7] and *“I skimmed it and went straight to the part of the document that I was most interested.”* [P8]

### Users of the guideline

When asked about who has used the guideline, survey respondents reported governance groups (*n* = 8), individuals (*n* = 6), and teams (*n* = 6), for example, project teams. Similarly, in interviews, participants described users of the guideline to be system builders and administrators, clinicians, and governance groups: *“When I read the NSW framework, it was really useful for builders, I think, and system administrators, especially if you’re maintaining the medication catalogue.”* [P3]

### Evaluation of the CDS guideline

#### Positive perceptions

When asked if the CDS guideline met expectations about what it should be providing, 11 of 14 survey respondents who answered the question agreed or strongly agreed (Figure 4).

**Figure 4.**
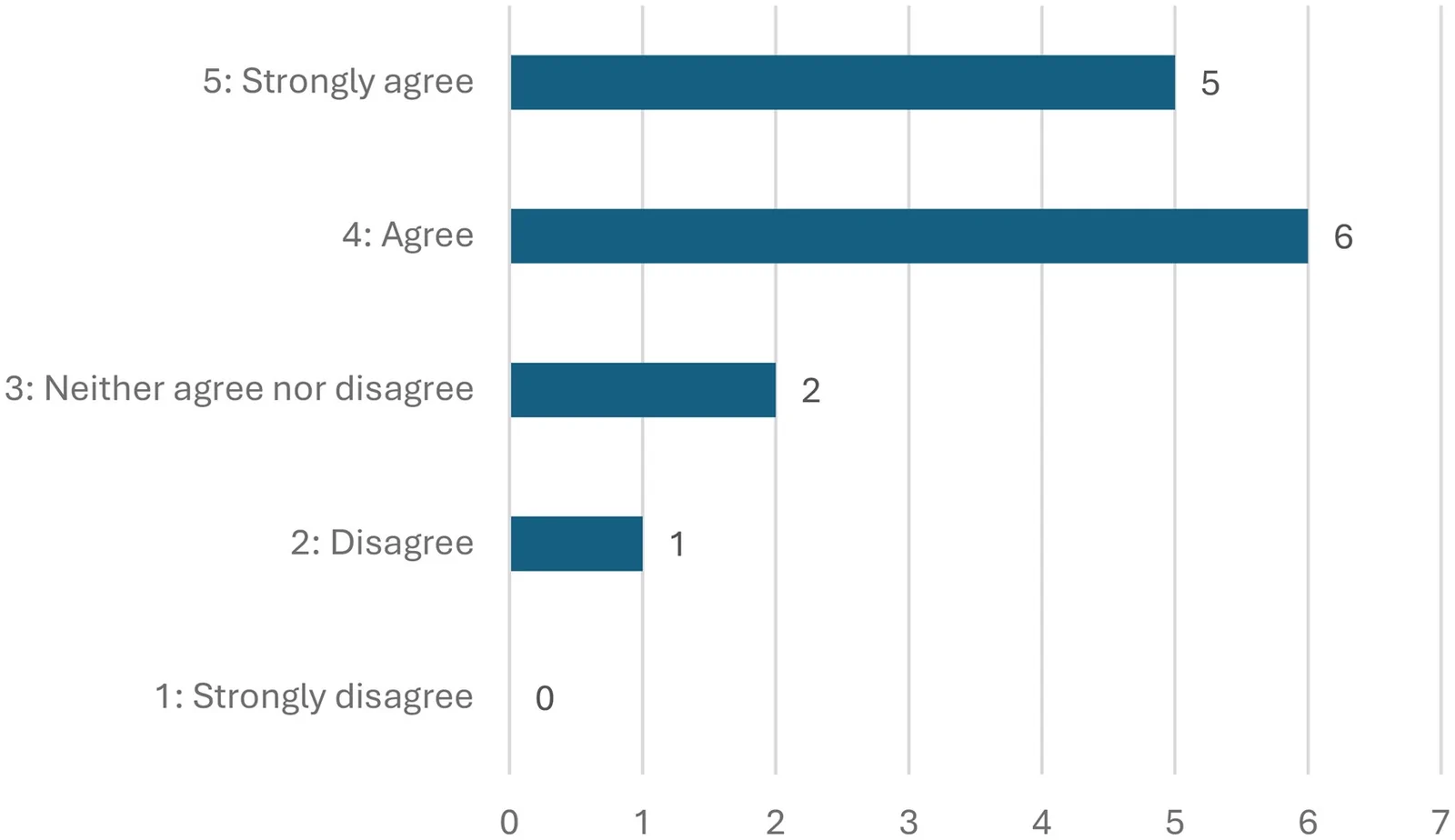
Respondents’ evaluation of whether the guideline met expectations.

When asked how likely they were to recommend the guideline to others on a scale of 1 to 10, the average score among survey respondents was 8.43 (range: 5–10).

Positive perceptions from survey respondents and interview participants generally included comments about the guideline’s usefulness and ease of use. Participants felt that the guideline was an improvement on current state: *“so much better than what we had before”* [PS15] and that it put focus on an important digital health element: *“focuses attention on an important aspect of digital health”* [PS24]. Participants indicated that the guideline filled a gap and first of its kind to offer practical and comprehensive guidance: *“one-stop guideline for CDS design”* [PS1], “*so that was where I think I referred to it a lot because I couldn’t find anything else as succinct and um and targeted to what I was looking for”* [P1] and *“I’m just excited about it…I have been trying to get a useful guide from anyone and anything …this work is so important and I think it’s the first work that I’ve ever seen that is actually practical, applicable and comprehensive. Like it’s just like this comprehensive package that I’m sure is just going to get better and better.”* [P4]

Table 2 provides a summary of features and characteristics of the guideline viewed positively by participants.

**Table 2.** Survey and interview participants’ positive perceptions of the features and characteristics of the guideline.

Positive perceptions	Quote/s from survey respondents (PS) and interview participants (P), where available
Content relating to passive CDS	*“it is great that there are clear examples of passive CDS”* [PS1]
Useful flowcharts, definitions, matrix, and tools	*“flowcharts, definitions and matrix have been the most useful”* [PS3] *“stepping out the 'How might we’ and '5 Whys’ methodology out loud with others was very helpful and something that I will continue to utilise” [PS17]* *“Oh, the alert rationalisation template was really, really useful…so the whole idea of filling in that form to really clarify to what your intent is when you’re trying to create an alert.”* [P3] *“I think what we really liked about it was first the algorithm about like risk stratification, like how high is the risk that you’re trying to mitigate and that will then tell you whether you need a passive or an interruptive alert.”* [P4] *“so you know we have now got a template for how an alert should be.”* [P7]
Vendor agnostic	*“I think the one thing that just kind of came to our mind, what we really loved…was that it was vendor agnostic. We liked that because we just switched from 1 vendor to the other.”* [P4]
Easy to use, good structure	*“easy to use”* [PS6] *“good structure…[that] considered all aspects”* [PS6]

Abbreviation: CDS, clinical decision support.

Overall, 13 of 14 (92.9%) survey respondents agreed or strongly agreed that the guide was useful overall, with sections 2 and 4 viewed as useful by all survey respondents (Appendix D). Ten of 14 (71.43%) respondents agreed or strongly agreed that the guideline was easy to use overall, and most respondents indicated that each section was also easy to use (Appendix D). The majority of survey respondents also indicated that the guideline, including its respective sections, was easy to understand and provided high satisfaction ratings. Appendix D contains further detail.

#### Perceived impacts of the guideline

Overall, participants cited that the guideline was impactful and provided benefits: *“the CDS Design Guideline has offered several notable benefits and positive impacts”* [PS22]. The majority of participants from both the survey and interviews found the guideline impactful in providing structure around CDS discussions, providing relevant evidence-based guidance around CDS, improving CDS decision making, providing confidence around CDS decision making, increasing knowledge/raising awareness of best practice thinking around CDS design, and challenging existing perceptions about what optimal CDS design looks like. Table 3 provides more detail on participant ratings of the guideline’s impact based on the survey and examples of corresponding quotes from the survey and interviews (where available).

**Table 3.** Participants’ ratings of the guideline’s impact and corresponding quotes from the survey and interviews.

Statement	Agreed or strongly agreed	Neither agreed or disagreed	Disagreed or strongly disagreed	Total
The guideline has been impactful in				

Providing structure around CDS discussions	12 (85.7%)	2 (14.3%)	0 (0%)	14

*“I do think that people kind of harp on and there’s like safety risks, there’s things that the system needs to do. And while I do agree that the system should support you to do the right thing, sometimes a lot that is subjective depending on the person you speak to and I think that having objective measures for how we’re actually making decisions around this is important.”* [P6] *“so I found it really useful as we work through some of the SDPR work on the [governance group] to have that guide to kind of encourage people along the decision-making process… so there’s a clear guide to follow when we’re making decisions”* [P9]				

Providing relevant evidence-based guidance around CDS	12 (85.7%)	1 (7.1%)	1 (7.1%)	14

*“you find that a lot of people are just making decisions in their own little pockets and silos and you end up with a lot of variation in the way that things are designed and implemented. But I guess if there could be some agreement on these like fundamental principles or you know, evidence based, you know, guidance then it would be a lot easier for those decisions to be made.”* [P1] “*it shows there’s a good evidence base behind it, which is the important part and I think it what? That’s what you really need is. You need an evidence based backing to say this is what we should be doing and why”* [P10] *“and it’s like you’re just saying it over and over again, but having something to point to that’s external to you…kind of makes it a little bit less personal and more, you know, authoritative. And there’s research behind this. This is not just me making stuff up or trying to be difficult.”* [P1]				

Improving CDS decision making	12 (85.7%)	2 (14.3%)	0 (0%)	14

*“I found as a chair, it’s been a really useful tool to have that guideline… I have a job to do to get a decision made that is in the best interest of the patients and the clinicians in NSW and we also have a guide to follow to support me in getting that done…So while the guide has been great in helping to decide the actual decision, it’s also been a really useful tool as a cat herder or chair to help with getting the decision-making going …”* [P9] *“it helped argue a case against implementing a nuisance alert that would not be sensitive enough, saving everyone involved a lot of time/reduction in alert fatigue”* [PS15]				

Providing confidence around CDS decision making	11 (78.6%)	3 (21.4%)	0 (0%)	14

*“I was confident with the information that I read that this has been recently reviewed of the literature. I’ve got something I don’t have to try to look everything up myself.”* [P3] *“I believe that users of the guideline are more confident in their decision-making, as it ensures that CDS tools are both reliable and evidence-based, leading to more accurate recommendations.”* [PS22]				

Increasing knowledge/raising awareness of best practice thinking around CDS design	12 (85.7%)	2 (14.3%)	0 (0%)	14

*“The structured approach outlined in the guideline has also fostered better collaboration between digital health analysts and clinicians, promoting a shared understanding of best practices when evaluating CDS”* [PS22]				

Challenging existing perceptions about what optimal CDS design looks like	12 (85.7%)	2 (14.3%)	0 (0%)	14

*“This document has been excellent at challenging perceptions about what good CDS should look like. It also provided advice about options other than alerts”* [PS19]. *“I think it was a very good way, particularly when there’s a little bit of a controversial alert.”* [P8] *“And so I think for us it was very exciting because even though kind of what I’d mentioned earlier didn’t give us all the granularity, it did make us realise if we had some blind spots…we should be looking more into this. And so that I think was just really great to have.”* [P4] *“I think we’ve evolved from slightly more [from] ad hoc finger in the air assessment of ‘is it a good idea to have a particular sort of like clinical decision support alert?’ to a more nuanced way to actually evaluate how whether or not things are actually appropriate within the system and looking at other ways we can actually support the clinical workflow.”* [P6]				

Abbreviations: CDS, clinical decision support; SDPR, Single Digital Patient Record.

#### Suggestions for improving the guideline

Table 4 summarizes suggestions for improving the guideline’s content and scope, and format and accessibility. Suggestions for improvement included addition of content, and making the guideline more accessible through templates and other formats. This was consistent with what was reported in interviews.

**Table 4.** Suggestions for improving the guideline.

Suggestions for improvement		
Area	Suggestion	Quote/s from the survey (PS) and interviews (P), where available
Content and scope	Add a glossary Include more CDS principles Develop a flowchart on how to use the document Include content on patient facing CDS Include examples or case studies Greater specificity on how to design specific elements	*“more principles for CDS rather than only the process for considering CDS inclusion”* [PS17] *“clarity on how to use the document ie, a flowchart on what section is useful for what’, and expanding the guideline’s scope to include patient facing CDS.”* [PS5] *“in my setting I would have liked some worked examples”* [P8] *“in terms of improvements moving forward, a bit more specificity around…specifically how to design things, whether it’s…things on the [Medication Administration Record] MAR, other things so you could sort of pick it up and apply directly and something around automation bias.”* [P4]
Format and accessibility	Develop simplified/cheat sheet versions of key flowcharts and tables Provide downloadable, fillable templates Make the guideline available via an app or interactive tool Break up information into smaller sections Include more guidance on how to use and navigate the document	*“consider abbreviated cheat sheets that could accompany the document.”* [PS1] *“I wanted a downloadable document with all the headings where info could be filled in about an alert and used by a governing body would have been helpful.”* [PS6] *“I would suggest it be developed as an interactive tool.”* [PS17] *“I would have liked like proformas or something to help me use it in that context.”* [P8] *“I’m just thinking if maybe that sort of bite size information might be easier to take in for some people”* [P1] *“maybe like flow charts or diagrams or something to help point people…to the right parts of the guideline, depending on what they’re trying to do.”* [P1]

Abbreviation: CDS, clinical decision support.

### Implementation strategies

Recommendations for improving the adoption and implementation of the guideline were provided by both survey and interview participants. These were grouped into 3 main themes: governance, training and engagement with users, and promotion of the guideline.

#### Governance

Survey respondents recommended that the guideline should be adopted by local (eg, Drugs and Therapeutics Committees) and state level governance committees to support decision making: *“Think it could and should be used by SQAG [the Safety and Quality Advisory Group], MSEAC [the Medication Safety Expert Advisory Committee], [and] ACI [the Agency for Clinical Innovation] when considering recommending eMeds changes/alerts or active decision support.”* [PS6].

Making use of the guideline mandatory as part of CDS governance intake processes was also mentioned: *“It should be mandatory to assess all CDS requests and new CDS tools against this guideline.”* [PS15] This was also recommended by interview participants: *“I think it should be a mandatory part of any application for design support and decision support. So if there’s a change request that comes through part of that checklist…you know the questions to ask to triage like that. There’s like a flow chart of, you know, what kind of support do we need for this kind of problem? So rather than having a request come through that says build this alert, it should be. This is the problem that we’re having. Let’s workshop it and figure out how to solve it using the most appropriate way. So I think that should be embedded in part of our design processes and our change request processes. Standardised form is probably the easiest way to do it. Make them make the clinicians tell us what they want, but also educate them at the same time in that form so that they’re thinking critically about what their desired outcome is at the end… work through the steps to what would be an acceptable solution to the problem? And don’t just jump to straight to ‘this is the alert and this is what I want it to say’ because I think that’s skipping a lot of the critical thinking steps and where we can use our human factors and human centered design expertise to do better…it needs to be embedded in a formal workflow.”* [P7].

#### Training and engagement with users of the guideline

Survey respondents suggested providing training and engaging with users of the guideline as another strategy to enhance the guideline’s implementation: *“I think sessions for relevant stakeholders would help keep them be informed about the guideline and any updates, as needed. Additionally, fostering collaborative feedback loops among stakeholders could ensure that necessary refinements are made to improve the guideline’s effectiveness, with continuous enhancements based on real-world data and feedback.”* [PS22] Likewise, interview participants also made suggestions to embed the guideline as a training tool to uplift capability around CDS: *“It’s great for as a training tool and an education tool for any new analysts coming on board.”* [P3]

#### Promotion of the guideline

Survey respondents recommended *“greater promotion of the guideline and referencing it in prepared materials in the e-health space”* [PS19] and prompting use of the guideline for broader system configuration, not just CDS decisions such as deciding on alerts. This was echoed by interview participants who recommended scaling up the guideline to a national or international level, raising further awareness of it, and making it more widely available and accessible: *“I think there’s definitely an opportunity to take it to a national level. And not redo the work because I think a lot of people would find it very useful.”* [P1], *“I’m just really grateful that you guys took the time and effort as NSW Health to create such a wonderful document that can be used Australia-wide potentially. Um, Victoria definitely doesn’t have anything similar, unfortunately, so it’s great that NSW are taking the leadership on this.”* [P3], and *“I do think it’s something that should be shared far and wide which is why I’ve been a championing it for our Interstate colleagues.”* [P7]

## Discussion

This study explored how a novel HF-informed CDS guideline was used in the context of real-world HIT projects. Feedback about the guideline was largely positive, with participants reporting that the guideline was easy to use, supported structured and evidence-based CDS decision making, and improved confidence in the selection and design of CDS. Users valued the guideline’s consolidated nature and step by step approach. Opportunities for improvement recommended by participants included incorporating additional practical tools, worked examples, and case studies to further support application across varied contexts.

As a general concept, guidelines and standards support the translation of evidence into consistent and repeatable practice.^20^^,^^21^ In the healthcare context, guidelines translate evidence into actionable recommendations to enhance healthcare delivery and outcomes.^22^ In our study, the majority of participants across both phases viewed the evidence-based nature of the guideline as a strength, and described how it supported discussions about CDS to reduce subjectivity and ensure decision making was guided by evidence. Critical appraisal instruments for guidelines, such as the Appraisal of Guidelines for Research and Evaluation, version II (AGREE II tool), suggest that the quality and effectiveness of a guideline can be assessed by its ability to link evidence to recommendations.^22^^,^^23^ Although some evidence-based guidelines, tools, and checklists to guide HIT design exist, these are not comprehensive repositories of evidence-based guidance for CDS design as they focus on specific aspects (eg, design of alerts^11^), and may be outdated (eg, Osheroff et al.’s 2012 guide^13^). Participants in our study commented on the guideline being a consolidation of the latest evidence and standards to produce actionable HF-informed, evidence-based recommendations and guidance for CDS designers. Participants reported that due to the evidence-based nature of the guideline, the guideline had a positive impact on confidence in CDS decision making, improved knowledge of best practice CDS design, enhanced CDS decision making, challenged ideas about CDS, guided the selection of CDS, and prevented the implementation of inappropriate CDS.

Although the content and usability of guidelines are important, implementation research suggests that the publication or dissemination of guidelines does not guarantee practice change.^24^ The uptake and use of guidelines within their context of use and associated processes are important determinants of effectiveness.^24^ Participants in our study indicated that the guideline informed processes, such as intake processes involving clinicians requesting new alerts, and was integrated into CDS decision-making governance structures. However, participants also recommended that further embedding the use of the guideline into existing workflows and the work of system designers would further enhance its uptake. These findings are consistent with other literature that purports that guideline effectiveness is influenced by its ability to be embedded at points of decision-making, and fit within workflows.^25–27^

A key finding from this study was that the HF-informed resource was seen to be effective in improving understanding and uplifting capability around the application of HF to optimize HF-informed CDS design. Carayon and Hoonakker^28^ argue that enhancing the practice of HF within the HIT context requires an understanding of the work system in which HIT is developed and implemented including the work of system designers. Our study revealed that system designers and those making decisions about CDS, as indicated by those who used the guideline, are primarily clinicians and informatics professionals. Furthermore, the study found that decision-making processes regarding CDS, although variable, generally involved decisions by individuals or teams and in many cases, multidisciplinary governance groups, consistent with other literature.^8^^,^^29^^,^^30^ The guideline was reported to be an effective strategy for integrating HF thinking and capability into the work of system designers, and other aspects of the work system in which CDS decisions are made.

Furthermore, it has been suggested that HF approaches must cater to the contextual realities and limitations of organizations responsible for designing HIT to be adopted sustainably.^9^ Participants within our study reported that they used the guideline flexibly based on needs, and reported variable uses for the guideline, from supporting discussions with individual clinicians to fully embedding the guideline as a framework for designing CDS within a large-scale HIT project. Therefore, the guideline appeared to provide flexible utility across a range of purposes and was used adaptably within the context real-world projects.

Our study provides several important implications for practice and real-world CDS design projects. As suggested by participants, the benefits of HF-informed CDS guidance are likely to be maximized when integrated into existing organizational workflows and governance processes. For example, the guideline could be incorporated into CDS request and intake processes to ensure stakeholders define the underlying problem and consider alternative interventions before requesting a specific solution such as an alert. Similarly, governance committees could use the guideline to support structured and evidence-based discussions when reviewing proposed CDS interventions. The guideline may also serve as a training and capability-building resource for new informatics professionals, analysts, system administrators, and clinicians involved in CDS decision-making, helping to establish a shared understanding of best-practice CDS design principles. Beyond prospective CDS design, organizations could also use the guideline during vendor system configuration and optimization activities to assess whether planned CDS interventions align with HF principles and local workflow requirements. The guideline may also support post-implementation monitoring and continuous improvement by providing a framework for reviewing existing CDS, identifying opportunities for optimization, and rationalizing ineffective or low-value interventions. Embedding the guideline within these routine activities may enhance adoption while supporting more consistent, transparent, and evidence-based CDS decision-making.

Participants also recommended mandating use of the guideline. This sentiment has been strongly echoed by others in the HIT academic literature and industry who advocate for formal requirements for organizations to routinely apply evidence-based CDS design guidance, embedded in governance, certification, and change processes.^31^^,^^32^ This approach would be akin to usability and human factors requirements mandated by the Food and Drug Administration (FDA) for device design.^8^^,^^31–33^ Making the CDS guideline more widely accessible nationally and internationally, as recommended by participants, would also assist with providing evidence-based, standardized approaches around CDS design on a larger scale, and avoid other organizations from duplicating efforts or re-inventing the wheel. Our study also identified several opportunities for continuous improvement of the guideline and its use in practice such as developing fillable templates and incorporating more case studies. This finding is also consistent with other literature which recommends that the application of evidence-based guidance should be supported by appropriate standardized tools and checklists.^8^^,^^31–33^

This study has several strengths. The 2-phased evaluation approach evaluated the guideline through the lens of usability domains including its effectiveness, ease of use, user satisfaction when applying the guideline, and usefulness.^11^^,^^18^ The evaluation approach also evaluated the guideline’s impact on CDS decision-making and the design of CDS. This combined process and outcome-based evaluation approach aligned with an evaluation framework which evaluates HF methods when applied to real-world projects and other evaluation approaches used to evaluate HF-informed HIT design tools.^7^^,^^11^ It also used a mixed-methods design, combining a survey (*n* = 25) with semi-structured interviews (*n* = 10), enabling both breadth and depth of insight. Importantly, the guideline was evaluated in the context of real-world use rather than hypothetical scenarios and interview data collection continued until thematic saturation was reached. However, several limitations should be acknowledged. The evaluation approach relied on subjective measures. Furthermore, the interview sample size was relatively small, although appropriate for qualitative inquiry and sufficient for saturation. Most interview participants were pharmacists working in informatics or digital health roles. While this may limit the transferability of findings to all potential users of the guideline, it is reflective of the context in which the study was conducted, where pharmacists commonly undertake informatics roles and are heavily involved in CDS governance, design, optimization, and implementation activities. Similarly, informatics professionals are typically the primary users of the guideline and are often responsible for reviewing CDS requests, supporting governance processes, and configuring CDS. As such, the perspectives captured are highly relevant to the intended audience of the guideline. However, the views of other stakeholder groups, including nurses, medical practitioners, health service managers, executives, vendor representatives, and system developers, may be underrepresented. Furthermore, several participants had experience with similar CDS projects, governance structures, or organizational initiatives, which may have contributed to the consistency of views expressed regarding the guideline’s usefulness and impact. Future studies should evaluate the guideline across a broader range of stakeholder groups, organizational contexts, and CDS projects to better understand differences in implementation and perceived impact. Most participants were based in Australia, with some international representation; however, the study did not explicitly examine differences across countries or organizational contexts, limiting insights into how contextual factors may influence the guideline’s use. Finally, the study did not directly assess outcomes related to uptake of the guideline, or the usability, safety, or effectiveness of CDS, relying instead on participant perceptions and self-reported experiences.

HF and systems thinking researchers purport that HF should not only inform the initial design of HIT but also support the ongoing design work that is required after implementation.^34^ As such, future evaluations of the guideline could delve into how it can be used to continuously improve and optimize CDS. Future research can also extend this work by incorporating more objective outcome measures, including usability testing, safety indicators, and CDS effectiveness metrics, to more comprehensively assess the impact of guideline on CDS usability and patient safety outcomes.

## Conclusion

This explanatory sequential mixed-methods study evaluated a consolidated, vendor agnostic, HF informed guideline for CDS design in the context of real-world HIT projects and use cases. Feedback on the guideline was largely positive with respect to its usefulness, ease of use, user satisfaction, and perceived impact on CDS decision making. Participants reported that the guideline helped structure and apply evidence to CDS decisions, while also creating a shared understanding of what good CDS design looks like across multidisciplinary teams and governance groups. The study also identified opportunities for enhancing the guideline including the addition of further practical tools, worked examples, and case studies to support application. This study is novel in that it represents the first evaluation of a consolidated HF‑informed CDS guideline used within real-world HIT projects. The findings suggest that embedding the use of HF informed CDS guidance can strengthen CDS governance and design processes. Future research should examine how such guidance supports ongoing CDS optimization and continuous improvement over time and incorporate more objective outcome measures related to CDS usability, safety, and effectiveness to more comprehensively assess impact.

## Supplementary Material

ocag138_Supplementary_Data

## Data Availability

Data used in this study are available upon reasonable request to the corresponding author, subject to data use agreements and ethics approval.
