# Supplementary material for: Evaluation of a clinical decision support design guideline
Source: J Am Med Inform Assoc. 2026 Aug 27;33(11):2114–26. doi: 10.1093/jamia/ocag138 (PMC13687750; doi:10.1093/jamia/ocag138)
Supplement: Supplementary file 1 [file ocag138_supplementary_data.zip › Evaluation of a CDS guideline - Appendix A - 240526.docx]

**Appendix A – Survey questions**

**Question**

**Demographics**

1. **Select the option which best describes your primary area of work while using the CDS Design Guideline**
   a. Healthcare provider
   b. Informatics/digital health
   c. Clinical governance / patient safety
   d. Department head/ manager
   e. Other: please describe

**Use of the CDS Design Guideline**

2. **Have you used the CDS Design Guideline? NB. For the purpose of answering this question, ‘use’ refers to any opportunity or situation where you have referred to the guideline.**
   a. Yes – continue to question 4
   b. No – continue to question 3,
3. **Can you please share your reasons for not using the CDS guideline, or any factors that prevented you from using it?**

[free text field for further explanation]

***Thank you for participating in our survey – end survey.***

4. **For what purpose/s have you used the CDS Design Guideline? Select all that apply and provide examples if possible:**
   a. Inform decision making about CDS
   b. Provide advice about CDS
   c. Support CDS discussions
   d. As an educational tool or reference material
   e. Other: please describe

[free text field for further explanation]

5. **How many use cases/situations have you used the CDS Design Guideline for?**
   a. 1
   b. 2-4
   c. 5-7
   d. More than 7
6. **Can you provide some details on how the CDS Design Guideline has been used by yourself or others in your organisation? Please select all that apply**
   a. **Who used the guideline?**
      i. Governance groups
      ii. Individuals
      iii. Teams e.g. project teams
      iv. Other: please describe
   b. **Which section/s have been used?**
      i. Section 1: About this document
      ii. Section 2: Determining the appropriateness of CDS and potential options
      iii. Section 3: CDS governance review
      iv. Section 4: Specific design guidance on CDS options
   c. **Please provide other relevant information on the guideline’s use e.g. how it was used, how long it took to use, resources required, etc**

[free text field for further explanation]

**Feedback on the CDS Design Guideline**

7. **The CDS Design Guideline met my expectations around what it should be providing:**

**1	2	3	4	5**

**Strongly disagree	Strongly agree**

[free text field for further explanation]

8. **I found the guideline useful (please rate each section of the guideline and the guideline overall):**
   a. Section 1: About this document

**1	2	3	4	5	N/A**

**Strongly disagree	Strongly agree**

b. Section 2: Determining the appropriateness of CDS and potential options

**1	2	3	4	5	N/A**

**Strongly disagree	Strongly agree**

c. Section 3: CDS governance review

**1	2	3	4	5	N/A**

**Strongly disagree	Strongly agree**

d. Section 4: Specific design guidance on CDS options

**1	2	3	4	5	N/A**

**Strongly disagree	Strongly agree**

e. The CDS Design Guideline overall

**1	2	3	4	5**

**Strongly disagree	Strongly agree**

9. **I found the guideline easy to use (please rate each section of the guideline and the guideline overall):**
   a. Section 1: About this document

**1	2	3	4	5	N/A**

**Strongly disagree	Strongly agree**

b. Section 2: Determining the appropriateness of CDS and potential options

**1	2	3	4	5	N/A**

**Strongly disagree	Strongly agree**

c. Section 3: CDS governance review

**1	2	3	4	5	N/A**

**Strongly disagree	Strongly agree**

d. Section 4: Specific design guidance on CDS options

**1	2	3	4	5	N/A**

**Strongly disagree	Strongly agree**

e. The CDS Design Guideline overall

**1	2	3	4	5**

**Strongly disagree	Strongly agree**

10. **I found the guideline easy to understand (please rate each section of the guideline and the guideline overall):**
    a. Section 1 About this document

**1	2	3	4	5	N/A**

**Strongly disagree	Strongly agree**

b. Section 2: Determining the appropriateness of CDS and potential options

**1	2	3	4	5	N/A**

**Strongly disagree	Strongly agree**

c. Section 3: CDS governance review

**1	2	3	4	5	N/A**

**Strongly disagree	Strongly agree**

d. Section 4: Specific design guidance on CDS options

**1	2	3	4	5	N/A**

**Strongly disagree	Strongly agree**

e. The CDS Design Guideline overall

**1	2	3	4	5**

**Strongly disagree	Strongly agree**

11. **I am satisfied with the guideline (please rate each section of the guideline and the guideline overall):**
    a. Section 1 About this document

**1	2	3	4	5	N/A**

**Strongly disagree	Strongly agree**

b. Section 2: Determining the appropriateness of CDS and potential options

**1	2	3	4	5	N/A**

**Strongly disagree	Strongly agree**

c. Section 3: CDS governance review

**1	2	3	4	5	N/A**

**Strongly disagree	Strongly agree**

d. Section 4: Specific design guidance on CDS options

**1	2	3	4	5	N/A**

**Strongly disagree	Strongly agree**

e. The CDS Design Guideline overall

**1	2	3	4	5**

**Strongly disagree	Strongly agree**

12. **On a scale from 0-10, how likely are you to recommend the CDS Design Guideline to others?**

**1	2	3	4	5	6	7	8	9	10**

13. **How can the CDS Design Guideline be improved?**

[free text field for further explanation]

**Impact of the CDS Design Guideline**

14. **The guideline has been impactful in:**
    i. providing structure around CDS discussions

**1	2	3	4	5**

**Strongly disagree	Strongly agree**

ii. providing relevant evidence-based guidance around CDS

**1	2	3	4	5**

**Strongly disagree	Strongly agree**

iii. improving CDS decision making

**1	2	3	4	5**

**Strongly disagree	Strongly agree**

iv. providing confidence around CDS decision making

**1	2	3	4	5**

**Strongly disagree	Strongly agree**

v. increasing knowledge/raising awareness of best practice thinking around CDS design

**1	2	3	4	5**

**Strongly disagree	Strongly agree**

vi. challenging existing perceptions about what optimal CDS design looks like

**1	2	3	4	5**

**Strongly disagree	Strongly agree**

15. **Please describe any other benefits or impacts of using the CDS Design Guideline that you have experienced.**

[free text field for further explanation]

**Implementation of the CDS Design Guideline**

16. **Can you suggest strategies to support current and future use/implementation of the CDS Design Guideline?**

[free text field for further explanation]
