# Supplementary material for: Evaluation of a clinical decision support design guideline
Source: J Am Med Inform Assoc. 2026 Aug 27;33(11):2114–26. doi: 10.1093/jamia/ocag138 (PMC13687750; doi:10.1093/jamia/ocag138)
Supplement: Supplementary file 1 [file ocag138_supplementary_data.zip › Evaluation of CDS guideline - Appendix B - 240526.docx]

**Appendix B – Interview questions**

1. Let’s start off with a few demographic questions. What position to you hold and please describe your role and general expertise. How long have you been in this position?
2. Have you used the CDS guideline in your organisation?
3. For all participants:
   a. Please describe how your organisation makes decision about CDS e.g. is there a governance group – who is involved, what are the processes in place? Perhaps use some examples to explain your processes.
   b. What do you like about your organisation’s CDS decision making and governance processes?
   c. What can be improved? And how?
   d. Are you confident in CDS decisions made by your organisation? Why or why not?
   e. What factors contribute to how CDS decisions are made?
   f. What does ‘effective CDS decision making’ look like to you?
   g. What factors influence whether decisions are made well or not well?
4. For users of the guideline:
   a. How has the guideline impacted your organisation’s CDS decision making and governance processes? How?
   b. Describe what you used the guideline for including what you did step by step to apply it? Who was involved in the application?
   c. Can you describe any learnings or reflections including any enablers and barriers to (what?)? What worked well and what didn’t?
   d. Can you describe any strategies that you have used or would recommend using to optimise use of the guideline during CDS decision making and governance processes?
   e. Would you use the guideline again? Why or why not?
