# Supplementary material for: Evaluation of a clinical decision support design guideline
Source: J Am Med Inform Assoc. 2026 Aug 27;33(11):2114–26. doi: 10.1093/jamia/ocag138 (PMC13687750; doi:10.1093/jamia/ocag138)
Supplement: Supplementary file 1 [file ocag138_supplementary_data.zip › Evaluation of CDS Guideline - Appendix C - 260526.docx]

**Appendix C – Respondents’ primary area of work when using the guideline**

| **Primary area of work** | **Number** | **Percentage (%)** |
| --- | --- | --- |
| Informatics/digital health | 11 | 45.8 |
| Clinical governance/patient safety | 5 | 20.8 |
| Healthcare provider | 3 | 12.5 |
| Department head/manager | 3 | 12.5 |
| Other: Academia | 1 | 4.2 |
| Other: Network Director | 1 | 4.2 |
| Total | 24 | 100 |
