# Supplementary material for: Evaluation of a clinical decision support design guideline
Source: J Am Med Inform Assoc. 2026 Aug 27;33(11):2114–26. doi: 10.1093/jamia/ocag138 (PMC13687750; doi:10.1093/jamia/ocag138)
Supplement: Supplementary file 1 [file ocag138_supplementary_data.zip › Evaluation of CDS guideline - Appendix D - 260526.docx]

**Appendix D – Participants’ ratings of usefulness, ease of use, ease of understanding and satisfaction**

| **Statement** | **Agreed or strongly agreed** | **Neither agreed or disagreed** | **Disagreed or strongly disagreed** | **Total** |
| --- | --- | --- | --- | --- |
| Usefulness | | | | |
| Overall I found the guideline useful | 13 (92.9%) | 1 (7.1%) | 0 (0%) | 14 |
| I found ‘Section 1: About this document’ useful | 8 (66.7%) | 0 (0%) | 4 (33.3%) | 12 |
| I found ‘Section 2: Determining the appropriateness of CDS and potential options’ useful | 13 (100%) | 0 (0%) | 0 (0%) | 13 |
| I found ‘Section 3: CDS governance review’ useful | 8 (72.7%) | 1 (9.1%) | 2 (18.2%) | 11 |
| I found ‘Section 4: Specific design guidance on CDS options’ useful | 12 (100%) | 0 (0%) | 0 (0%) | 12 |
| Ease of use | | | | |
| Overall I found the guideline easy to use | 10 (71.4%) | 1 (7.1%) | 3 (21.4%) | 14 |
| I found ‘Section 1: About this document’ easy to use | 8 (72.7%) | 1 (9.1%) | 2 (18.2%) | 11 |
| I found ‘Section 2: Determining the appropriateness of CDS and potential options’ easy to use | 11 (84.6%) | 0 (0%) | 2 (15.4%) | 13 |
| I found ‘Section 3: CDS governance review’ easy to use | 8 (88.9%) | 0 (0%) | 1 (11.1%) | 9 |
| I found ‘Section 4: Specific design guidance on CDS options’ easy to use | 11 (91.7%) | 0 (0%) | 1 (8.3%) | 12 |
| Easy to understand | | | | |
| Overall I found the guideline easy to understand | 11 (78.6%) | 2 (14.3%) | 1 (7.1%) | 14 |
| I found ‘Section 1: About this document’ easy to understand | 10 (83.3%) | 2 (16.7%) | 0 (0%) | 12 |
| I found ‘Section 2: Determining the appropriateness of CDS and potential options’ easy to understand | 12 (92.3%) | 1 (7.7%) | 0 (0%) | 13 |
| I found ‘Section 3: CDS governance review’ easy to understand | 8 (80.0%) | 2 (20.0%) | 0 (0%) | 10 |
| I found ‘Section 4: Specific design guidance on CDS options’ easy to understand | 11 (91.7%) | 1 (8.3%) | 0 (0%) | 12 |
| Satisfaction | | | | |
| Overall I am satisfied with the guideline | 11 (78.6%) | 3 (21.4%) | 0 (0%) | 14 |
| I am satisfied with ‘Section 1: About this document’ | 9 (75.0%) | 2 (16.7%) | 1 (8.3%) | 12 |
| I am satisfied with ‘Section 2: Determining the appropriateness of CDS and potential options’ | 10 (76.9%) | 3 (23.1%) | 0 (0%) | 13 |
| I am satisfied with ‘Section 3: CDS governance review’ | 9 (90.0%) | 1 (10.0%) | 0 (0%) | 10 |
| I am satisfied with ‘Section 4: Specific design guidance on CDS options’ | 11 (91.7%) | 1 (8.3%) | 0 (0%) | 12 |
